# Photonic time crystals

**DOI:** 10.1038/s41598-017-17354-6

**Published:** 2017-12-07

**Authors:** Lunwu Zeng, Jin Xu, Chengen Wang, Jianhua Zhang, Yuting Zhao, Jing Zeng, Runxia Song

**Affiliations:** 1 College of Engineering, Nanjing Agricultural University, Nanjing, 210031 China; 2 Sydney University, Sydney, Australia

## Abstract

When space (time) translation symmetry is spontaneously broken, the space crystal (time crystal) forms; when permittivity and permeability periodically vary with space (time), the photonic crystal (photonic time crystal) forms. We proposed the concept of photonic time crystal and rewritten the Maxwell’s equations. Utilizing Finite Difference Time Domain (FDTD) method, we simulated electromagnetic wave propagation in photonic time crystal and photonic space-time crystal, the simulation results show that more intensive scatter fields can obtained in photonic time crystal and photonic space-time crystal.

## Introduction

Time independent systems possess continuous time translation symmetry, which is the most fundamental symmetry, and the spontaneous breaking of time translation symmetry leads to time crystal. The concept of the time crystal was first proposed by Wilczek and Shapere^1,2^, then Li *et al*. conducted the experiment, realized space-time crystal by using trapped ions in a ring threaded by an Aharonov-Bohm flux^3^. Subsequent work^4^ revealed that the experiment^3^ could not explain the existence of the time crystal, and the time crystal was forbidden in equilibrium^4,5^. Since then, many researchers have made further investigations. They found that the time crystal exists again in non-equilibrium Floquet systems because of a loophole^6–17^. Under the condition of periodical-driven, the discrete time crystal (or Floquet time crystal) in non-equilibrium Floquet systems could form which broke time translation symmetry^8^, the period of discrete time crystal is the integer multiple of the drive period^10,11^. Meanwhile, utilizing completely different quantum system, Choi *et al*.^12^ and Zhang *et al*.^13^ experimentally observed the same significant features of a time crystal state: oscillations at integer multiple of the drive period. To form a time crystal by spontaneous breaking time translation symmetry is similar to form space crystal by spontaneous breaking space translation symmetry. Similar to space crystal in condensate matter, the photonic space crystal was proposed in electromagnetic system^18,19^, we proposed photonic time crystal and photonic space-time crystal in electromagnetic system based on time crystal in condensate matter, and simulated electromagnetic wave propagation in one-dimensional (1D), two-dimensional (2D), three-dimensional (3D) photonic time crystal and photonic space-time crystal.

## Results

### Electromagnetic wave propagates in 1D photonic time crystal

Electromagnetic wave propagation is described by the Maxwell’s equation, in photonic time crystal, permittivity and permeability are the function of time, which vary with time periodically, for free source case, the Maxwell’s equations $\nabla \times \overrightarrow{E}(\overrightarrow{r},t)\,=$ $-\partial ({\mu }_{0}{\mu }_{r}(\overrightarrow{r},t)\overrightarrow{H}(\overrightarrow{r},t))/\partial t$ and $\nabla \times \overrightarrow{H}(\overrightarrow{r},t)=\partial ({\varepsilon }_{0}{\varepsilon }_{r}(\overrightarrow{r},t)\overrightarrow{E}(\overrightarrow{r},t))/\partial t$ can be rewritten as

1 $$\nabla \times \overrightarrow{E}(\overrightarrow{r},t)=-{\mu }_{0}\frac{\partial {\mu }_{r}(\overrightarrow{r},t)}{\partial t}\overrightarrow{H}(\overrightarrow{r},t)-{\mu }_{0}{\mu }_{r}(\overrightarrow{r},t)\frac{\partial \overrightarrow{H}(\overrightarrow{r},t)}{\partial t}.$$

2 $$\nabla \times \overrightarrow{H}(\overrightarrow{r},t)={\varepsilon }_{0}\frac{\partial {\varepsilon }_{r}(\overrightarrow{r},t)}{\partial t}\overrightarrow{E}(\overrightarrow{r},t)+{\varepsilon }_{0}{\varepsilon }_{r}(\overrightarrow{r},t)\frac{\partial \overrightarrow{E}(\overrightarrow{r},t)}{\partial t}$$

where $\overrightarrow{E}(\overrightarrow{r},t)$ and $\overrightarrow{H}(\overrightarrow{r},t)$ are the time harmonic electric field and time harmonic magnetic field, respectively, *ε*_0_ and *μ*_0_ are the permittivity and permeability in free space, respectively. ${\varepsilon }_{r}(\overrightarrow{r},t)$ and ${\mu }_{r}(\overrightarrow{r},t)$ are time- and space-dependent relative permittivity and permeability, respectively. Utilizing FDTD method^20^, Eqs (1) and (2) can be discretized. For 1D photonic time crystal, ∂/∂*x* = 0, ∂/∂*y* = 0, the discrete electric field and magnetic field can be written as (see Supplementary Information)

3 $${{\rm{E}}}_{{\rm{x}}}^{{\rm{n}}+1}(k)=\frac{{\varepsilon }^{n}(k)}{{\varepsilon }^{n+1}(k)}{{\rm{E}}}_{{\rm{x}}}^{{\rm{n}}}(k)-\frac{{\rm{\Delta }}t}{{\varepsilon }^{n+1}(k)}\frac{{{\rm{H}}}_{{\rm{y}}}^{{\rm{n}}+\tfrac{1}{2}}(k+\tfrac{1}{2})-{{\rm{H}}}_{{\rm{y}}}^{{\rm{n}}+\tfrac{1}{2}}(k-\tfrac{1}{2})}{{\rm{\Delta }}z}$$

4 $${{\rm{H}}}_{{\rm{y}}}^{{\rm{n}}+\tfrac{1}{2}}(k+\tfrac{1}{2})=\frac{{\mu }^{n-\tfrac{{\rm{1}}}{{\rm{2}}}}(k+\tfrac{1}{2})}{{\mu }^{{\rm{n}}+\tfrac{1}{2}}(k+\tfrac{1}{2})}{{\rm{H}}}_{{\rm{y}}}^{{\rm{n}}-\tfrac{1}{2}}(k+\tfrac{1}{2})-\frac{{\rm{\Delta }}t}{{\mu }^{n+\tfrac{1}{2}}(k+\tfrac{1}{2})}\frac{{{\rm{E}}}_{{\rm{x}}}^{{\rm{n}}}(k+1)-{{\rm{E}}}_{{\rm{x}}}^{{\rm{n}}}(k)}{{\rm{\Delta }}z}$$

where *t* = (*n* + 1/2)Δ*t*, Δ*t* is time step, *n* is total number of time step, *k* is the position of grid cell. The permittivity in any grid cell is equal at same time, so *ε* ^*n*^(*k* + 1/2) = *ε* ^*n*^(*k*) = …, so is the permeability in any grid cell, it can be written as ${\mu }^{n}(k+1/2)={\mu }^{n}(k)=\mathrm{...}$.

**Figure 1:**
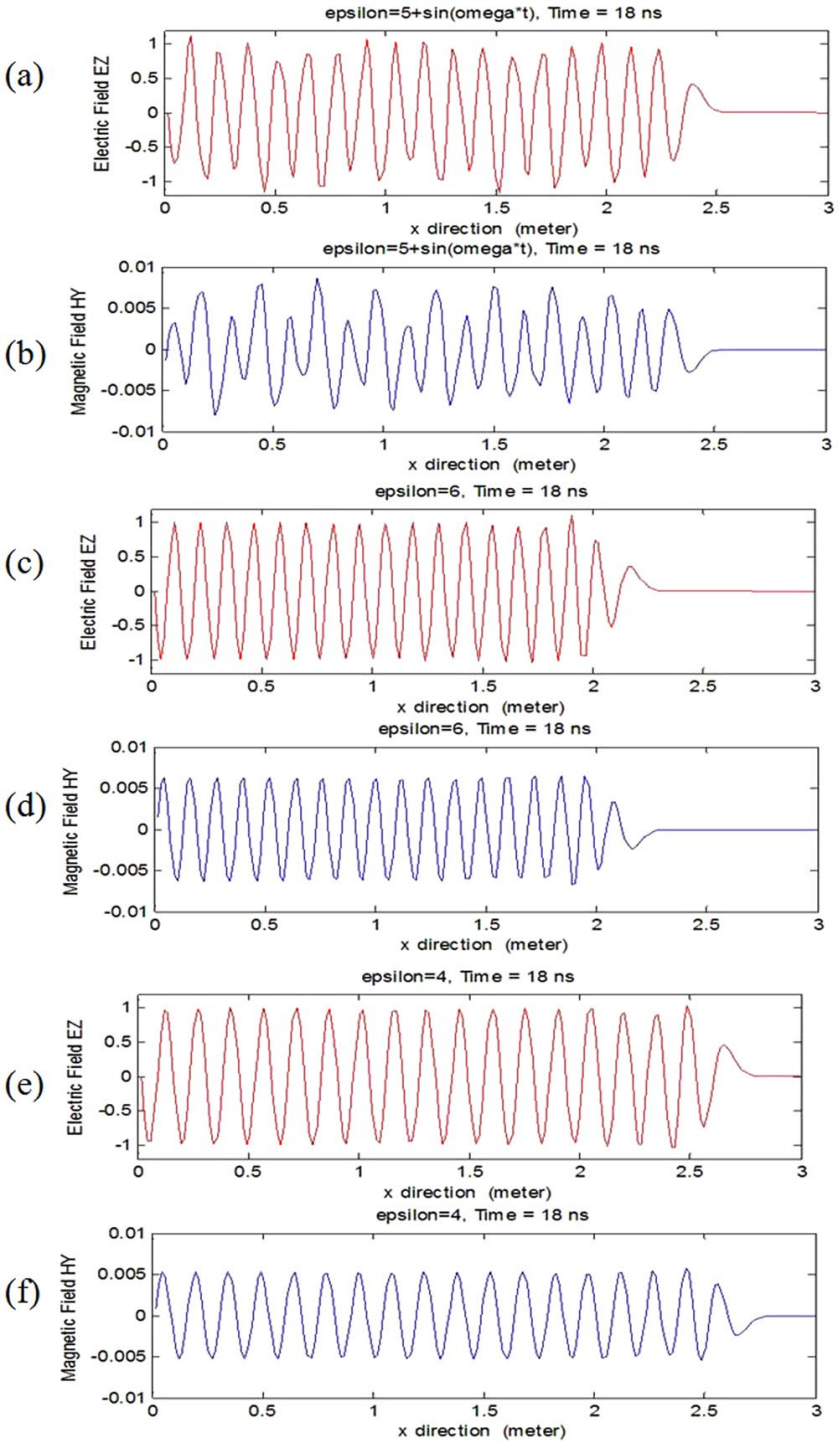
Electromagnetic wave propagates in 1D photonic time crystal and photonic non-time crystal (conventional dielectric). (**a**) and (**b**) Electric and magnetic field propagates in 1D photonic time crystal, *ε*(*t*) = 5 + sin *ω*t, the amplitudes vary with space and time. (**c**) and (**d**) Electric and magnetic field propagates in 1D photonic non-time crystal, *ε*(*t*) = 6, the amplitudes are invariant. (**e**) and (**f**) Electric and magnetic field propagates in 1D photonic non-time crystal, *ε*(*t*) = 4, the amplitudes are also invariant. The propagation time is at 18 ns.

In Fig. 1, we simulated electromagnetic wave propagation in 1D photonic time crystal and photonic non-time crystal (conventional dielectric), the total number of grid cell is 200, the space increment is 0.015 m, the total number of time step is 360, the time step is 5 × 10^−11^ *s*. To simplify, suppose that the relative permittivity varies with time periodically and relative permeability is a constant, namely, the relative permittivity and permeability of the photonic time crystal are *ε*(*t*) = 5 + sin *ωt* and *μ*(*t*) = 1, respectively, Fig. 1a and b show the electromagnetic wave propagation in 1D photonic time crystal, the source is a sine signal, *E*_z0_ = sin *ωt*, *ω* = 2*πf*, the frequency *f* = 1.0 × 10^9^ *Hz*, the amplitudes vary with space and time. For comparison, we simulated electromagnetic wave propagation in 1D photonic non-time crystal in Fig. 1c and d, the relative permittivity and permeability of the photonic non-time crystal are *ε*(*t*) = 6 ≥ 5 + sin *ωt* and *μ*(*t*) = 1, respectively, the amplitudes are invariant. Figure 1e and f show the electromagnetic wave propagation in 1D photonic non-time crystal, the relative permittivity and permeability are *ε*(*t*) = 4 ≤ 5 + sin *ωt* and *μ*(*t*) = 1, respectively, the amplitudes are invariant too. We concluded that in the photonic time crystal, the impedances don’t match with each other, electromagnetic wave is scattered everywhere, so the amplitudes vary with space and time, the amplitudes in photonic time crystal (Fig. 1a and b) are smaller than those in photonic non-time crystal (Fig. 1c–f).

### Electromagnetic wave scattered by a 2D photonic time crystal

**Figure 2:**
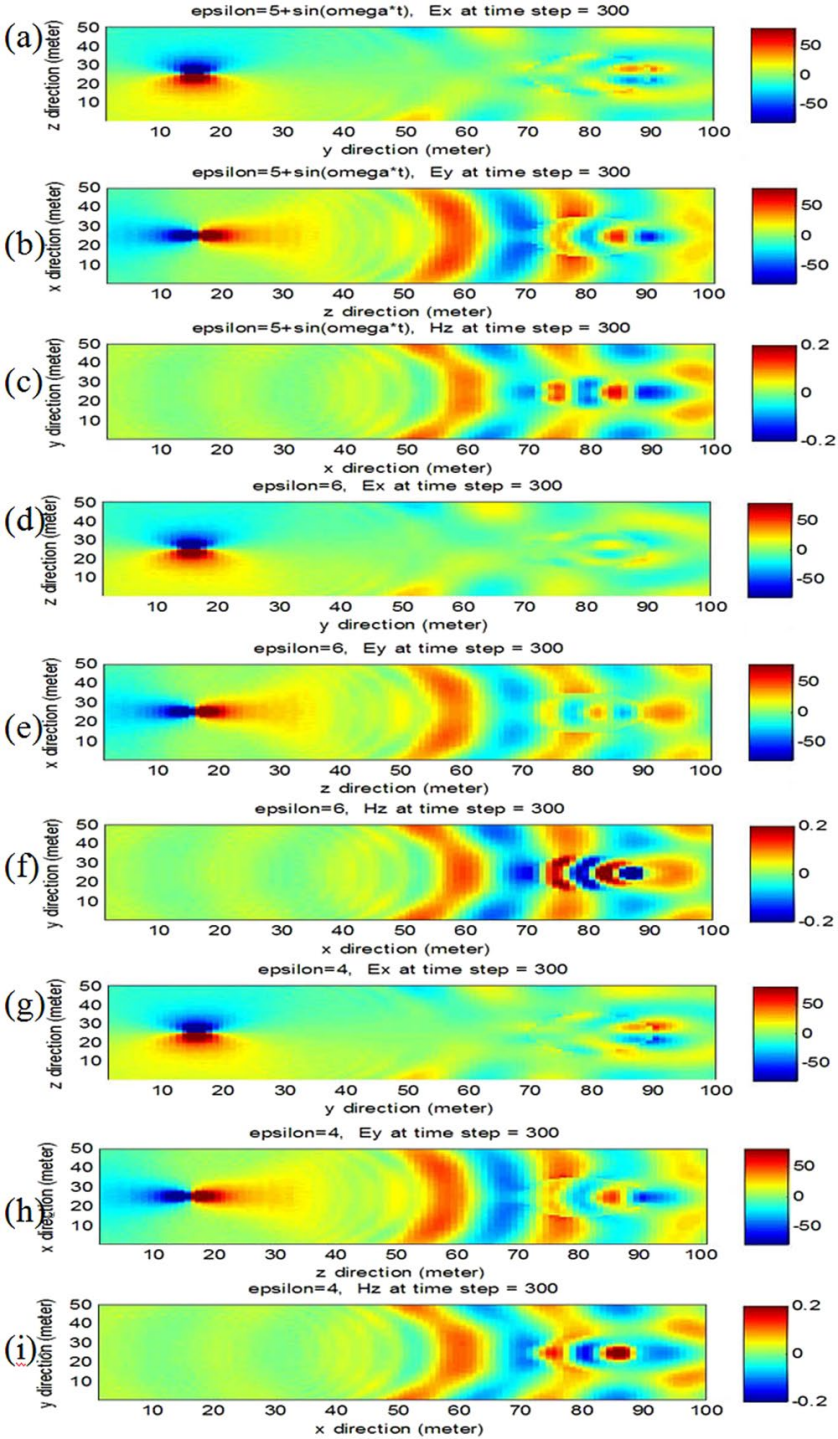
Electromagnetic wave scattered by 2D photonic time crystal and photonic non-time crystal cylinder. (**a**–**c**) Electric and magnetic field scattered by 2D photonic time crystal cylinder, *ε*(*t*) = 5 + sin *ω*t. (**d**–**f**) Electric and magnetic field scattered by 2D photonic non-time crystal cylinder, *ε*(*t*) = 6. (**g**–**i**) Electric and magnetic field scattered by 2D photonic time crystal cylinder, *ε*(*t*) = 4. The scatter time is at time step 300, namely, 1.5 ns.

For 2D photonic time crystal (TM wave and TE wave), ∂/∂*z* = 0, the discrete electric field and magnetic field can be obtained (see Supplementary Information). In Fig. 2, we simulated the electromagnetic wave scattered by a 2D photonic time crystal and 2D photonic non-time crystal cylinder, the total number of grid cell is x = 100, y = 50, the space increment is Δx = Δy = 0.003 m, the total number of time step is 300, the time step is 5.0 × 10^−12^ s, the radius of the cylinder is 0.01 m, the center of the cylinder is located at x = 80, y = 25. Figure 2a–c show the electromagnetic wave scattered by a 2D photonic time crystal cylinder, the relative permittivity and permeability of the cylinder are *ε*(*t*) = 5 + sin *ω*t and *μ*(*t*) = 1, respectively, the source is also a sine signal, *H*_z0_ = *A*sin *ωt*, the frequency *f* = 5.0 × 10^9^ *Hz*. For comparison, Fig. 2d–f show the electromagnetic wave scattered by a 2D photonic non-time crystal cylinder, the relative permittivity and permeability of the cylinder are *ε*(*t*) = 6 and *μ*(*t*) = 1, respectively. Figure 2g–i show the electromagnetic wave scattered by a 2D photonic non-time crystal cylinder, the relative permittivity and permeability of the cylinder are *ε*(*t*) = 4 and *μ*(*t*) = 1, respectively.

### Electromagnetic wave propagates in 3D photonic time crystal

**Figure 3:**
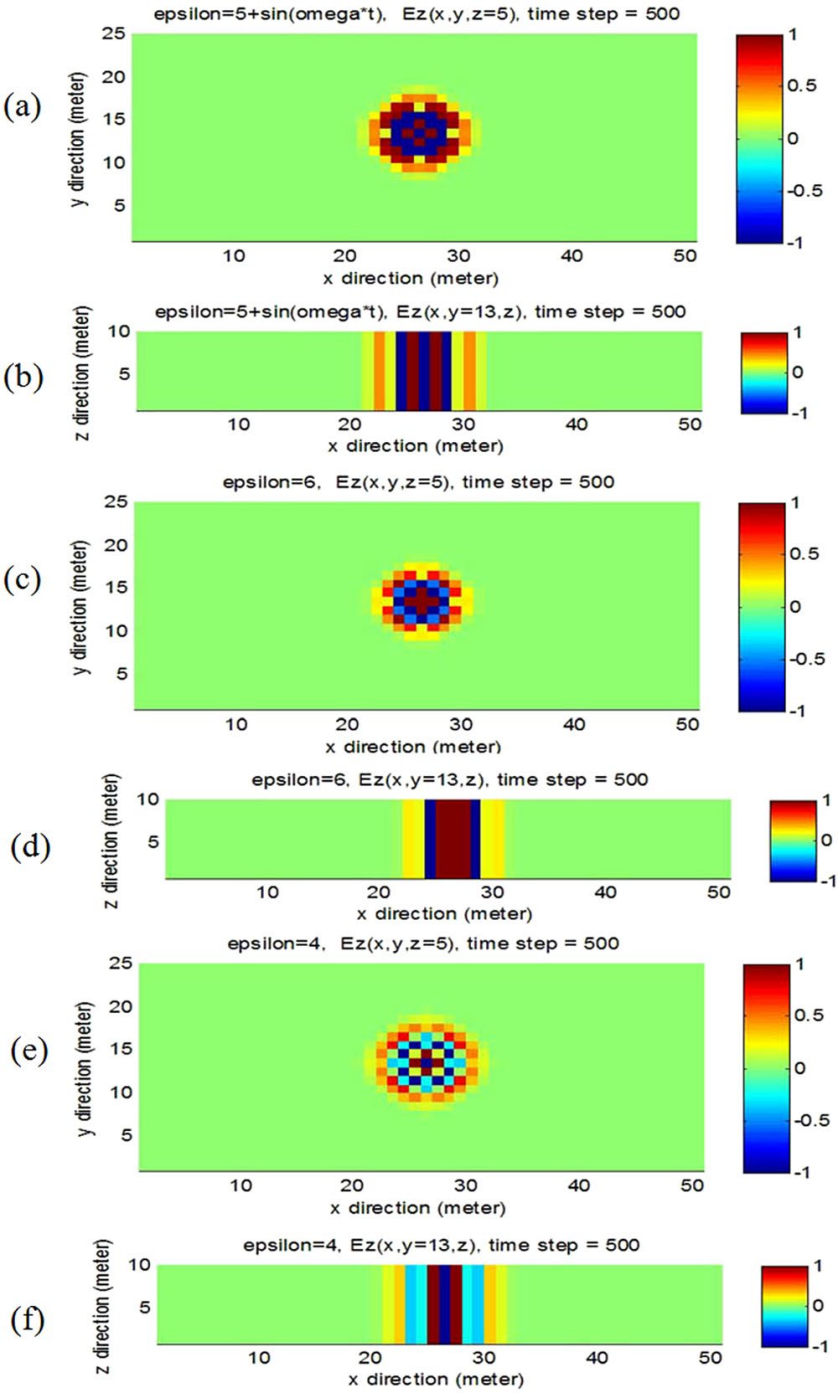
Electromagnetic wave propagates in 3D photonic time crystal and 3D photonic non-time crystal. (**a**) Electric field propagates in 3D photonic time crystal, seeing in z-direction, *ε*(*t*) = 5 + sin *ωt*. (**b**) Electric field propagates in 3D photonic time crystal, seeing in y-direction, *ε*(*t*) = 5 + sin *ωt*. (**c**) Electric field propagates in 3D photonic non-time crystal, seeing in z-direction, *ε*(*t*) = 6. (**d**) Electric field propagates in 3D photonic non-time crystal, seeing in y-direction, *ε*(*t*) = 6. (**e**) Electric field propagates in 3D photonic non-time crystal, seeing in z-direction, *ε*(*t*) = 4. (**f**) Electric field propagates in 3D photonic non-time crystal, seeing in y-direction, *ε*(*t*) = 4. The propagation time is at the time step 500.

**Figure 4:**
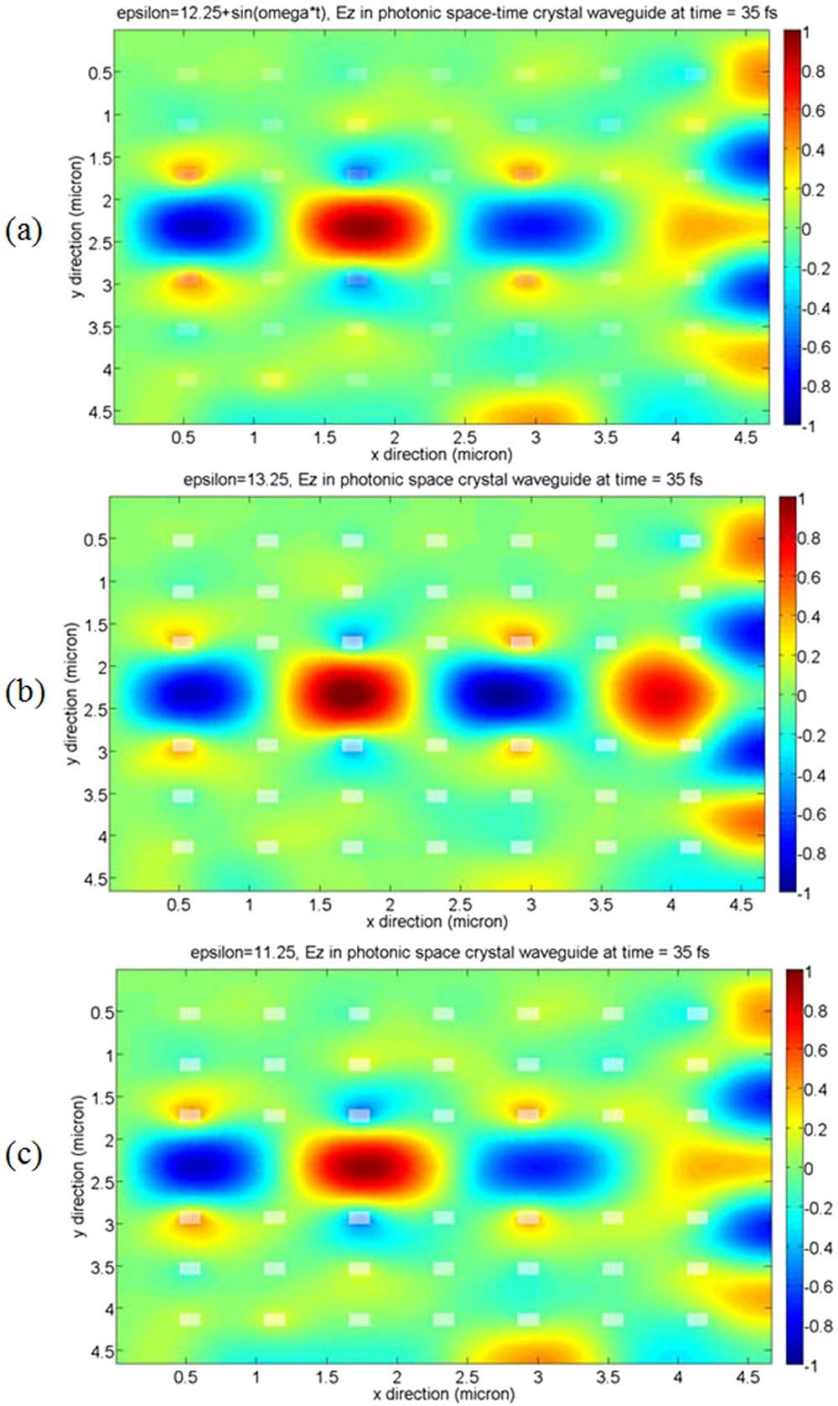
Electromagnetic wave propagates in 2D photonic space-time crystal and photonic space crystal. (**a**) Electromagnetic wave propagates in 2D photonic space-time crystal, the permittivity of the periodical array dielectrics is *ε*(*t*) = 12.25 + sin *ω*t. (**b**) Electromagnetic wave propagates in 2D photonic space crystal, the permittivity of the periodical array dielectrics is *ε*(*t*) = 13.25. (**c**) Electromagnetic wave propagates in 2D photonic space crystal, the permittivity of the periodical array dielectrics is *ε*(*t*) = 11.25. The propagation time is at the time step 400.

For 3D photonic time crystal, the discrete electric field and magnetic field can be obtained (see Supplementary Information). In Fig. 3, we simulated electromagnetic wave propagation in 3D photonic time crystal and photonic non-time crystal, the total number of grid cell in three direction is x = 50, y = 24, z = 10, correspondingly, the space increment Δx = Δy = Δz = 200 *m*, the total number of time step is 500, the time step is 3.3 × 10^−12^ s. Figure 3a and b show electromagnetic wave propagation in 3D photonic time crystal, the relative permittivity and permeability of the photonic time crystal are *ε*(*t*) = 5 + sin *ω*t and *μ*(*t*) = 1, respectively, the frequency of the source is *f* = 1.0 × 10^9^ *Hz*. For comparison, Fig. 3c and d show the electromagnetic wave propagation in 3D photonic non-time crystal, the relative permittivity and permeability of the photonic non-time crystal are *ε*(*t*) = 6 and *μ*(*t*) = 1, respectively. Figure 4e and f show the electromagnetic wave propagation in 3D photonic non-time crystal, the relative permittivity and permeability of the photonic non-time crystal are *ε*(*t*) = 4 and *μ*(*t*) = 1, respectively.

### Electromagnetic wave propagates in photonic space-time crystal

In Fig. 4, we simulated electromagnetic wave propagation in 2D photonic space-time crystal (the permittivity of the periodical array dielectrics varies with time periodically in photonic space crystal) and photonic space crystal. The photonic crystals consist of 7 × 7 periodical array dielectrics surrounded with air, the side length is 1.5 × 10^−7^ *m*. The relative permittivity of the air is *ε* = 1. A central horizontal line of seven periodical structures are removed to form a central wave guide. The space increment Δx = Δy = 1.5 × 10^−8^ *m*, the total number of time step is 1000, the time step is 3.5 × 10^−17^ s. Figure 4a shows electromagnetic wave propagation in 2D photonic space-time crystal, the relative permittivity and permeability of the period array dielectrics are *ε*(*t*) = 12.25 + sin *ω*t and *μ*(*t*) = 1, respectively, the frequency of the source is *f* = 1.9 × 10^14^ *Hz*. For comparison, Fig. 4b shows the electromagnetic wave propagation in 2D photonic space crystal, the relative permittivity and permeability of the periodical array dielectrics are *ε*(*t*) = 13.25 and *μ*(*t*) = 1, respectively. Figure 4c shows the electromagnetic wave propagation in 2D photonic space crystal, the relative permittivity and permeability of the periodical array dielectrics are *ε*(*t*) = 11.25 and *μ*(*t*) = 1, respectively. The periodical array dielectrics in Fig. 4a are darker than those in Fig. 4b and c, this is because the permittivity of the periodical array dielectrics is not uniform in photonic space-time crystal, and the scatter fields in photonic space-time crystal are more intensive than those in photonic space crystal.

**Figure 5:**
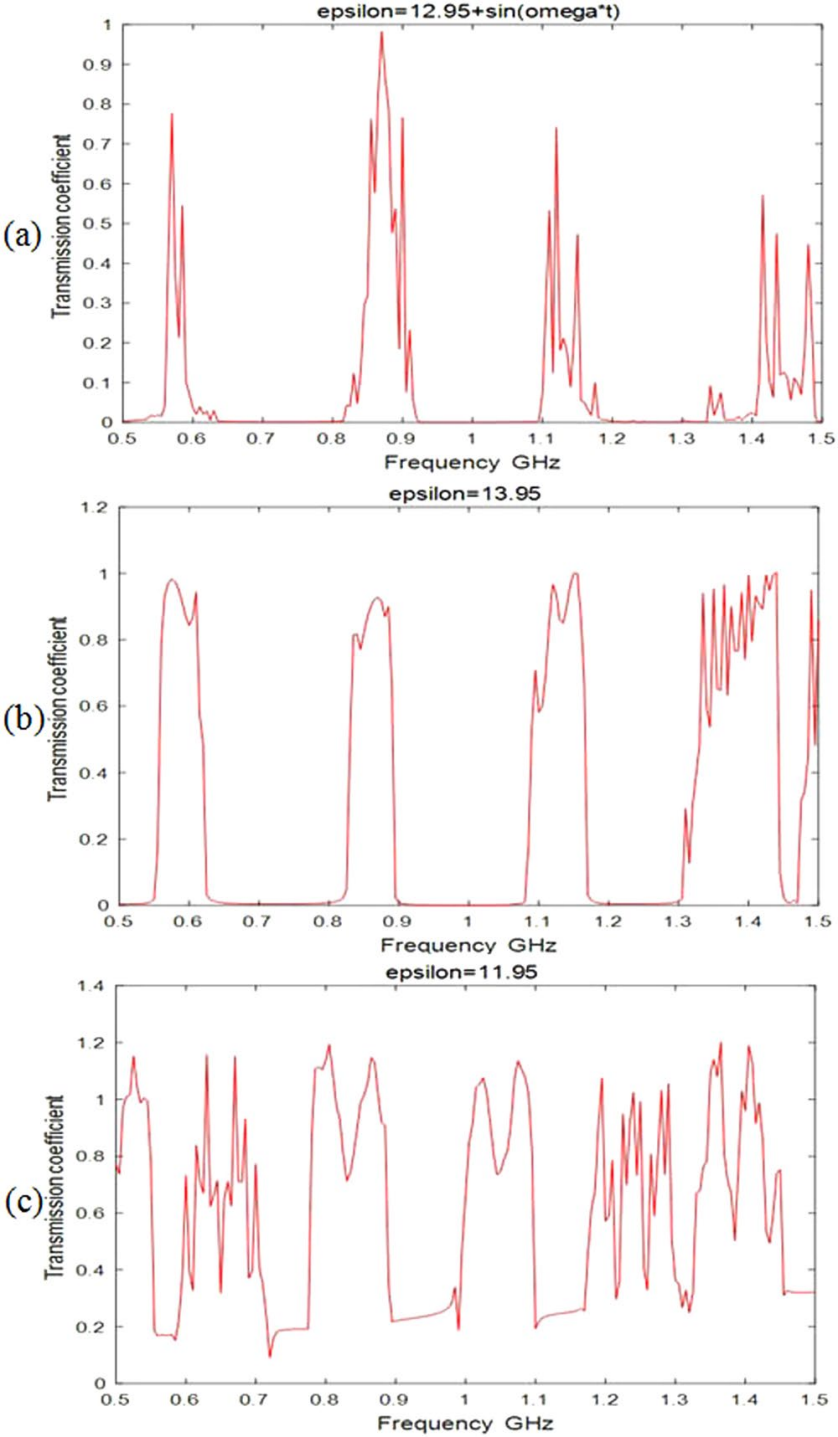
Electromagnetic wave propagates in 2D photonic space-time crystal and photonic space crystal, the relation between the transmission coefficient and the frequency. (**a**) The permittivity of the periodical array dielectrics is *ε*(*t*) = 12.25 + sin *ωt*. (**b**) The permittivity of the periodical array dielectrics is *ε*(*t*) = 13.25. (**c**) The permittivity of the periodical array dielectrics is *ε*(*t*) = 11.25.

In Fig. 5, we simulated electromagnetic wave propagation in 2D photonic space-time crystal and photonic space crystal. Figure 5a shows the relation between transmission coefficient and the frequency in 2D photonic space-time crystal, the permittivity and permeability of the periodical array dielectrics are *ε*(*t*) = 12.95 + sin *ω*t and *μ*(*t*) = 1, respectively. Figure 5b shows the relation between transmission coefficient and the frequency in 2D photonic space crystal, the permittivity and permeability of the periodical array dielectrics are *ε*(*t*) = 13.95 and *μ*(*t*) = 1, respectively. Figure 5c also shows the relation between transmission coefficient and the frequency in 2D photonic space crystal, the permittivity and permeability of the periodical array dielectrics are *ε*(*t*) = 11.95 and *μ*(*t*) = 1, respectively. The band gaps in Fig. 5a are larger than those in Fig. 5b and c, this is because the permittivity of the periodical array dielectrics in photonic space-time crystal is not uniform, and the scatter fields in photonic space-time crystal are more intensive than those in photonic space crystal, namely, the larger band gaps can be obtained in photonic space-time crystal.

Theoretically, the field-dependent dielectric^21–23^ can be designed as a photonic time crystal, yet, in high frequency, the permittivity varying with time is not obvious^23^, it is very difficult to make the period of electromagnetic field equal to that of permittivity. As for some heat diffusion materials, such as silicon and germanium, the heat conductivity, mass density and specific heat vary with temperature, by adjusting temperature periodically, one might make the period of the temperature field equal to that of material parameters. Whereas, for some acoustic wave materials, one can also adjust mass density and bulk module periodically to design acoustic time crystal. By the same method, other time crystals, like mass diffusion time crystal, could be designed too. It should be pointed out that reference^8,12,13^ described the discrete time crystal whose period is the integer multiple of the drive period and robustness against external perturbations. Wilczek and Shapere^1,2^ stressed the periodical movement in the lowest energy state of the time crystal. Whereas, our research focused on the permittivity and permeability of photonic time crystal varying with time periodically. Borzdov studied electromagnetic space-time crystal of a quantum version^24,25^, however, all physically observable properties of quantum systems are time independent in equilibrium, so no time-periodic behavior can manifest itself^11^.

## Conclusion

In this work, we proposed the concept of the photonic time crystal and photonic space-time crystal, and simulated electromagnetic wave propagation in 1D, 2D, 3D photonic time crystal and photonic space-time crystal, the simulated results indicate that the scatter fields in photonic time crystal are more intensive than those in photonic non-crystal, and the band gaps in photonic time crystal are larger than those in photonic space crystal. The method we adopted provides the possibility for further investigation in other time crystal and space-time crystal.

## Methods

In photonic time crystal, the Maxwell’s equations can be rewritten as in Eqs (1) and (2). Utilizing FDTD method^20^, Eqs (1) and (2) can be discretized. For one-dimensional photonic time crystal, ∂/∂*x* = 0, ∂/∂*y* = 0, Eqs (1) and (2) can be discretized as Eqs (3) and (4). For two-dimensional photonic time crystal of TM wave, ∂/∂*z* = 0, Eqs (1) and (2) can be discretized as

5 $${{\rm{H}}}_{{\rm{x}}}^{{\rm{n}}+\tfrac{{\rm{1}}}{{\rm{2}}}}(i,j+\tfrac{1}{2})=\frac{{\mu }^{n-\tfrac{1}{2}}(i,j+\tfrac{1}{2})}{{\mu }^{n+\tfrac{1}{2}}(i,j+\tfrac{1}{2})}{{\rm{H}}}_{{\rm{x}}}^{{\rm{n}}-\tfrac{{\rm{1}}}{{\rm{2}}}}(i,j+\tfrac{1}{2})-\frac{{\rm{\Delta }}t}{{\mu }^{n+\tfrac{1}{2}}(i,j+\tfrac{1}{2})}\frac{{{\rm{E}}}_{{\rm{z}}}^{{\rm{n}}}(i,j+1)-{{\rm{E}}}_{{\rm{z}}}^{{\rm{n}}}(i,j)}{{\rm{\Delta }}y}$$

6 $${{\rm{H}}}_{{\rm{y}}}^{{\rm{n}}+\tfrac{{\rm{1}}}{{\rm{2}}}}(i+\tfrac{1}{2},j)=\frac{{\mu }^{n-\tfrac{1}{2}}(i+\tfrac{1}{2},j)}{{\mu }^{n+\tfrac{1}{2}}(i+\tfrac{1}{2},j)}{{\rm{H}}}_{{\rm{y}}}^{{\rm{n}}-\tfrac{{\rm{1}}}{{\rm{2}}}}(i+\tfrac{1}{2},j)+\frac{{\rm{\Delta }}t}{{\mu }^{n+\tfrac{1}{2}}(i+\tfrac{1}{2},j)}\frac{{{\rm{E}}}_{{\rm{z}}}^{{\rm{n}}}(i+1,j)-{{\rm{E}}}_{{\rm{z}}}^{{\rm{n}}}(i,j)}{{\rm{\Delta }}x}$$

7 $$\begin{array}{rcl}{{\rm{E}}}_{{\rm{z}}}^{{\rm{n}}+{\rm{1}}}(i,j) & = & \frac{{\varepsilon }^{n}(i,j)}{{\varepsilon }^{n+1}(i,j)}{{\rm{E}}}_{{\rm{z}}}^{{\rm{n}}}(i,j)+\frac{{\rm{\Delta }}t}{{\varepsilon }^{n+1}(i,j)}\\  &  & \times (\frac{{{\rm{H}}}_{{\rm{y}}}^{{\rm{n}}+\tfrac{{\rm{1}}}{{\rm{2}}}}(i+\tfrac{1}{2},j)-{{\rm{H}}}_{{\rm{y}}}^{{\rm{n}}+\tfrac{{\rm{1}}}{{\rm{2}}}}(i-\tfrac{1}{2},j)}{{\rm{\Delta }}x}\\  &  & -\frac{{{\rm{H}}}_{{\rm{x}}}^{{\rm{n}}+\tfrac{{\rm{1}}}{{\rm{2}}}}(i,j+\tfrac{1}{2})-{{\rm{H}}}_{{\rm{x}}}^{{\rm{n}}+\tfrac{{\rm{1}}}{{\rm{2}}}}(i,j-\tfrac{1}{2})}{{\rm{\Delta }}y})\end{array}$$

For TE wave, ∂/∂*z* = 0, Eqs (1) and (2) can be discretized as

8 $$\begin{array}{rcl}{{\rm{E}}}_{{\rm{x}}}^{{\rm{n}}+1}(i+\tfrac{1}{2},j) & = & \frac{{\varepsilon }^{n}(i+\tfrac{1}{2},j)}{{\varepsilon }^{n+1}(i+\tfrac{1}{2},j)}{{\rm{E}}}_{{\rm{x}}}^{{\rm{n}}}(i+\tfrac{1}{2},j)\\  &  & +\,\frac{{\rm{\Delta }}t}{{\varepsilon }^{n+1}(i+\tfrac{1}{2},j)}\frac{{{\rm{H}}}_{{\rm{z}}}^{{\rm{n}}+\tfrac{1}{2}}(i+\tfrac{1}{2},j+\tfrac{1}{2})-{{\rm{H}}}_{{\rm{z}}}^{{\rm{n}}+\tfrac{1}{2}}(i+\tfrac{1}{2},j-\tfrac{1}{2})}{{\rm{\Delta }}y}\end{array}$$

9 $$\begin{array}{rcl}{{\rm{E}}}_{{\rm{y}}}^{{\rm{n}}+1}(i,j+\tfrac{1}{2}) & = & \frac{{\varepsilon }^{n}(i,j+\tfrac{1}{2})}{{\varepsilon }^{n+1}(i,j+\tfrac{1}{2})}{{\rm{E}}}_{{\rm{y}}}^{{\rm{n}}}(i,j+\tfrac{1}{2})\\  &  & +\,\frac{{\rm{\Delta }}t}{{\varepsilon }^{n+1}(i,j+\tfrac{1}{2})}\frac{{{\rm{H}}}_{{\rm{z}}}^{{\rm{n}}+\tfrac{1}{2}}(i+\tfrac{1}{2},j+\tfrac{1}{2})-{{\rm{H}}}_{{\rm{z}}}^{{\rm{n}}+\tfrac{1}{2}}(i-\tfrac{1}{2},j+\tfrac{1}{2})}{{\rm{\Delta }}x}\end{array}$$

10 $$\begin{array}{rcl}{{\rm{H}}}_{{\rm{z}}}^{{\rm{n}}+\tfrac{1}{2}}(i+\tfrac{1}{2},j+\tfrac{1}{2}) & = & \frac{{\mu }^{n-\tfrac{{\rm{1}}}{{\rm{2}}}}(i+\tfrac{1}{2},j+\tfrac{1}{2})}{{\mu }^{{\rm{n}}+\tfrac{1}{2}}(i+\tfrac{1}{2},j+\tfrac{1}{2})}{{\rm{H}}}_{{\rm{z}}}^{{\rm{n}}-\tfrac{1}{2}}(i+\tfrac{1}{2},j+\tfrac{1}{2})\\  &  & -\,\frac{{\rm{\Delta }}t}{{\mu }^{n+\tfrac{1}{2}}(i+\tfrac{1}{2},j+\tfrac{1}{2})}(\frac{{{\rm{E}}}_{{\rm{y}}}^{{\rm{n}}}(i+1,j+\tfrac{1}{2})-{{\rm{E}}}_{{\rm{y}}}^{{\rm{n}}}(i,j+\tfrac{1}{2})}{{\rm{\Delta }}x}\\  &  & -\,\frac{{{\rm{E}}}_{{\rm{x}}}^{{\rm{n}}}(i+\tfrac{1}{2},j+1)-{{\rm{E}}}_{{\rm{x}}}^{{\rm{n}}}(i+\tfrac{1}{2},j)}{{\rm{\Delta }}y})\end{array}$$

For three-dimensional photonic time crystal, Eqs (1) and (2) can be discretized as

11 $$\begin{array}{rcl}{{\rm{E}}}_{{\rm{x}}}^{{\rm{n}}+1}(i+\tfrac{1}{2},j,k) & = & \frac{{\varepsilon }^{n}(i+\tfrac{1}{2},j,k)}{{\varepsilon }^{n+1}(i+\tfrac{1}{2},j,k)}{{\rm{E}}}_{{\rm{x}}}^{{\rm{n}}}(i+\tfrac{1}{2},j,k)\\  &  & +\frac{{\rm{\Delta }}t}{{\varepsilon }^{n+1}(i+\tfrac{1}{2},j,k)}\frac{{{\rm{H}}}_{{\rm{z}}}^{{\rm{n}}+\tfrac{1}{2}}(i+\tfrac{1}{2},j+\tfrac{1}{2},k)-{{\rm{H}}}_{{\rm{z}}}^{{\rm{n}}+\tfrac{1}{2}}(i+\tfrac{1}{2},j-\tfrac{1}{2},k)}{{\rm{\Delta }}y}\\  &  & -\frac{{\rm{\Delta }}t}{{\varepsilon }^{n+1}(i+\tfrac{1}{2},j,k)}\frac{{{\rm{H}}}_{{\rm{y}}}^{{\rm{n}}+\tfrac{1}{2}}(i+\tfrac{1}{2},j,k+\tfrac{1}{2})-{{\rm{H}}}_{{\rm{y}}}^{{\rm{n}}+\tfrac{1}{2}}(i+\tfrac{1}{2},j,k-\tfrac{1}{2})}{{\rm{\Delta }}z}\end{array}$$

12 $$\begin{array}{rcl}{{\rm{E}}}_{{\rm{y}}}^{{\rm{n}}+1}(i,j+\tfrac{1}{2},k) & = & \frac{{\varepsilon }^{n}(i,j+\tfrac{1}{2},k)}{{\varepsilon }^{n+1}(i,j+\tfrac{1}{2},k)}{{\rm{E}}}_{{\rm{x}}}^{{\rm{n}}}(i,j+\tfrac{1}{2},k)\\  &  & +\frac{{\rm{\Delta }}t}{{\varepsilon }^{n+1}(i,j+\tfrac{1}{2},k)}\frac{{{\rm{H}}}_{{\rm{x}}}^{{\rm{n}}+\tfrac{1}{2}}(i,j+\tfrac{1}{2},k+\tfrac{1}{2})-{{\rm{H}}}_{{\rm{x}}}^{{\rm{n}}+\tfrac{1}{2}}(i,j+\tfrac{1}{2},k-\tfrac{1}{2})}{{\rm{\Delta }}z}\\  &  & -\frac{{\rm{\Delta }}t}{{\varepsilon }^{n+1}(i,j+\tfrac{1}{2},k)}\frac{{{\rm{H}}}_{{\rm{z}}}^{{\rm{n}}+\tfrac{1}{2}}(i+\tfrac{1}{2},j+\tfrac{1}{2},k)-{{\rm{H}}}_{{\rm{z}}}^{{\rm{n}}+\tfrac{1}{2}}(i-\tfrac{1}{2},j+\tfrac{1}{2},k)}{{\rm{\Delta }}x}\end{array}$$

13 $$\begin{array}{rcl}{{\rm{E}}}_{{\rm{z}}}^{{\rm{n}}+1}(i,j,k+\tfrac{1}{2}) & = & \frac{{\varepsilon }^{n}(i,j,k+\tfrac{1}{2})}{{\varepsilon }^{n+1}(i,j,k+\tfrac{1}{2})}{{\rm{E}}}_{{\rm{z}}}^{{\rm{n}}}(i,j,k+\tfrac{1}{2})\\  &  & +\frac{{\rm{\Delta }}t}{{\varepsilon }^{n+1}(i,j,k+\tfrac{1}{2})}\frac{{{\rm{H}}}_{{\rm{y}}}^{{\rm{n}}+\tfrac{1}{2}}(i+\tfrac{1}{2},j,k+\tfrac{1}{2})-{{\rm{H}}}_{{\rm{y}}}^{{\rm{n}}+\tfrac{1}{2}}(i-\tfrac{1}{2},j,k+\tfrac{1}{2})}{{\rm{\Delta }}x}\\  &  & -\frac{{\rm{\Delta }}t}{{\varepsilon }^{n+1}(i,j,k+\tfrac{1}{2})}\frac{{{\rm{H}}}_{{\rm{x}}}^{{\rm{n}}+\tfrac{1}{2}}(i,j+\tfrac{1}{2},k+\tfrac{1}{2})-{{\rm{H}}}_{{\rm{x}}}^{{\rm{n}}+\tfrac{1}{2}}(i,j-\tfrac{1}{2},k+\tfrac{1}{2})}{{\rm{\Delta }}y}\end{array}$$

14 $$\begin{array}{rcl}{{\rm{H}}}_{{\rm{x}}}^{{\rm{n}}+\tfrac{1}{2}}(i,j+\tfrac{1}{2},k+\tfrac{1}{2}) & = & \frac{{\mu }^{n-\tfrac{1}{2}}(i,j+\tfrac{1}{2},k+\tfrac{1}{2})}{{\mu }^{n+\tfrac{1}{2}}(i,j+\tfrac{1}{2},k+\tfrac{1}{2})}{{\rm{H}}}_{{\rm{x}}}^{{\rm{n}}-\tfrac{1}{2}}(i,j+\tfrac{1}{2},k+\tfrac{1}{2})\\  &  & -\frac{{\rm{\Delta }}t}{{\mu }^{n+\tfrac{1}{2}}(i,j+\tfrac{1}{2},k+\tfrac{1}{2})}\frac{{{\rm{E}}}_{{\rm{z}}}^{{\rm{n}}}(i,j+1,k+\tfrac{1}{2})-{E}_{{\rm{z}}}^{{\rm{n}}}(i,j,k+\tfrac{1}{2})}{{\rm{\Delta }}y}\\  &  & +\frac{{\rm{\Delta }}t}{{\mu }^{n+\tfrac{1}{2}}(i,j+\tfrac{1}{2},k+\tfrac{1}{2})}\frac{{{\rm{E}}}_{{\rm{y}}}^{{\rm{n}}}(i,j+\tfrac{1}{2},k+1)-{E}_{{\rm{y}}}^{{\rm{n}}}(i,j+\tfrac{1}{2},k)}{{\rm{\Delta }}z}\end{array}$$

15 $$\begin{array}{rcl}{{\rm{H}}}_{{\rm{y}}}^{{\rm{n}}+\tfrac{1}{2}}(i+\tfrac{1}{2},j,k+\tfrac{1}{2}) & = & \frac{{\mu }^{n-\tfrac{1}{2}}(i+\tfrac{1}{2},j,k+\tfrac{1}{2})}{{\mu }^{n+\tfrac{1}{2}}(i+\tfrac{1}{2},j,k+\tfrac{1}{2})}{{\rm{H}}}_{{\rm{y}}}^{{\rm{n}}-\tfrac{1}{2}}(i+\tfrac{1}{2},j,k+\tfrac{1}{2})\\  &  & -\frac{{\rm{\Delta }}t}{{\mu }^{n+\tfrac{1}{2}}(i+\tfrac{1}{2},j,k+\tfrac{1}{2})}\frac{{{\rm{E}}}_{{\rm{x}}}^{{\rm{n}}}(i+\tfrac{1}{2},j,k+1)-{E}_{{\rm{x}}}^{{\rm{n}}}(i+\tfrac{1}{2},j,k)}{{\rm{\Delta }}z}\\  &  & +\frac{{\rm{\Delta }}t}{{\mu }^{n+\tfrac{1}{2}}(i+\tfrac{1}{2},j,k+\tfrac{1}{2})}\frac{{{\rm{E}}}_{{\rm{z}}}^{{\rm{n}}}(i+1,j,k+\tfrac{1}{2})-{E}_{{\rm{z}}}^{{\rm{n}}}(i,j,k+\tfrac{1}{2})}{{\rm{\Delta }}x}\end{array}$$

16 $$\begin{array}{rcl}{{\rm{H}}}_{{\rm{z}}}^{{\rm{n}}+\tfrac{1}{2}}(i+\tfrac{1}{2},j+\tfrac{1}{2},k) & = & \frac{{\mu }^{n-\tfrac{1}{2}}(i+\tfrac{1}{2},j+\tfrac{1}{2},k)}{{\mu }^{n+\tfrac{1}{2}}(i+\tfrac{1}{2},j+\tfrac{1}{2},k)}{{\rm{H}}}_{{\rm{z}}}^{{\rm{n}}-\tfrac{1}{2}}(i+\tfrac{1}{2},j+\tfrac{1}{2},k)\\  &  & -\frac{{\rm{\Delta }}t}{{\mu }^{n+\tfrac{1}{2}}(i+\tfrac{1}{2},j+\tfrac{1}{2},k)}\frac{{{\rm{E}}}_{{\rm{y}}}^{{\rm{n}}}(i+1,j+\tfrac{1}{2},k)-{E}_{{\rm{y}}}^{{\rm{n}}}(i,j+\tfrac{1}{2},k)}{{\rm{\Delta }}x}\end{array}$$

The discretized methods are shown in Supplementary Information, according to the discretized results, we simulated electromagnetic field propagation in photonic time crystal and photonic space-time crystal by Matlab software.

## Electronic supplementary material

Supplementary Information
