# Supplementary material for: Photonic time crystals
Source: Sci Rep. 2017 Dec 7;7:17165. doi: 10.1038/s41598-017-17354-6 (PMC5719460; doi:10.1038/s41598-017-17354-6)
Supplement: Supplementary file 1 — Supplementary Information [file 41598_2017_17354_MOESM1_ESM.pdf]

Supplementary Information for "Photonic time crystal"

Lunwu Zeng[1], Jin Xu[1], Chengen Wang[1], Jianhua Zhang[1], Yuting Zhao[1], Jing Zeng[2] and Runxia Song[1]

[1]*College of Engineering, Nanjing Agricultural University, Nanjing 210031, China*

[2]*Sydney University, Sydney, Australia*

A1. One-dimensional photonic time crystal

Electromagnetic wave propagation are described by the Maxwell's equation, in photonic time crystal, permittivity and permeability are the function of time, and periodically vary with time, for free source case, the Maxwell's equations $\nabla\times\vec{E}(\vec{r},t)=-\partial(\mu_0\mu_r(\vec{r},t)\vec{H}(\vec{r},t))/\partial t$ and $\nabla\times\vec{H}(\vec{r},t)=\partial(\varepsilon_0\varepsilon_r(\vec{r},t)\vec{E}(\vec{r},t))/\partial t$ can be rewritten as

$$\nabla\times\vec{E}(\vec{r},t)=-\mu_0\frac{\partial\mu_r(\vec{r},t)}{\partial t}\vec{H}(\vec{r},t)-\mu_0\mu_r(\vec{r},t)\frac{\partial\vec{H}(\vec{r},t)}{\partial t}, \tag{A1}$$

$$\nabla\times\vec{H}(\vec{r},t)=\varepsilon_0\frac{\partial\varepsilon_r(\vec{r},t)}{\partial t}\vec{E}(\vec{r},t)+\varepsilon_0\varepsilon_r(\vec{r},t)\frac{\partial\vec{E}(\vec{r},t)}{\partial t}, \tag{A2}$$

where $\vec{E}(\vec{r},t)$ and $\vec{H}(\vec{r},t)$ are the time harmonic electric field and time harmonic magnetic field, respectively, ε_0 and μ_0 are the permittivity and permeability in free space, respectively, In photonic space crystal, $\varepsilon_r(\vec{r},t)$ and $\mu_r(\vec{r},t)$ are the space-dependent relative permittivity and permeability, respectively. In photonic time crystal, $\varepsilon_r(\vec{r},t)$ and $\mu_r(\vec{r},t)$ are the time-dependent relative permittivity and permeability, respectively. In photonic space-time crystal, $\varepsilon_r(\vec{r},t)$ and $\mu_r(\vec{r},t)$ are the time- and space-dependent relative permittivity and permeability, respectively.

Utilizing FDTD method [1], Eq. (A1) and Eq. (A2) can be discretized. For one-dimensional photonic time crystal, $\partial/\partial x = 0$, $\partial/\partial y = 0$, Eq. (A1) and Eq. (A2) can be written as

$$-\frac{\partial \mathrm{H}_y(\vec{r},t)}{\partial z} = \varepsilon(\vec{r},t)\frac{\partial \mathrm{E}_x(\vec{r},t)}{\partial t} + \frac{\partial \varepsilon(\vec{r},t)}{\partial t}\mathrm{E}_x(\vec{r},t) \tag{A3}$$

$$\frac{\partial \mathrm{E}_x(\vec{r},t)}{\partial z} = -\mu(\vec{r},t)\frac{\partial \mathrm{H}_y(\vec{r},t)}{\partial t} - \frac{\partial \mu(\vec{r},t)}{\partial t}\mathrm{H}_y(\vec{r},t) \tag{A4}$$

According to Fig. A1, the discrete electric field and magnetic field are

$$\mathrm{E}_x^{n+1}(k) = \frac{\varepsilon^n(k)}{\varepsilon^{n+1}(k)}\mathrm{E}_x^n(k) - \frac{\Delta t}{\varepsilon^{n+1}(k)}\frac{\mathrm{H}_y^{n+\frac{1}{2}}(k+\frac{1}{2}) - \mathrm{H}_y^{n+\frac{1}{2}}(k-\frac{1}{2})}{\Delta z} \tag{A5}$$

$$\mathrm{H}_y^{n+\frac{1}{2}}(k+\tfrac{1}{2}) = \frac{\mu^{n-\frac{1}{2}}(k+\frac{1}{2})}{\mu^{n+\frac{1}{2}}(k+\frac{1}{2})}\mathrm{H}_y^{n-\frac{1}{2}}(k+\tfrac{1}{2}) - \frac{\Delta t}{\mu^{n+\frac{1}{2}}(k+\frac{1}{2})}\frac{\mathrm{E}_x^n(k+1) - \mathrm{E}_x^n(k)}{\Delta z} \tag{A6}$$

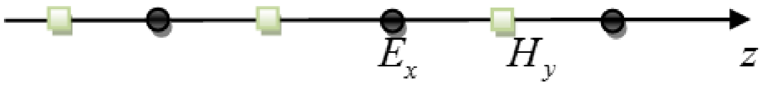

Fig. A1. One-dimensional Yee cell.

A2. Two-dimensional photonic time crystal

For two-dimensional photonic time crystal (TM wave), $\partial/\partial z = 0$, Eq. (A1) and Eq. (A2) can be written as

$$\frac{\partial \mathrm{E}_z(\vec{r},t)}{\partial y} = -\mu(\vec{r},t)\frac{\partial \mathrm{H}_x(\vec{r},t)}{\partial t} - \frac{\partial \mu(\vec{r},t)}{\partial t}\mathrm{H}_x(\vec{r},t) \tag{A7}$$

$$\frac{\partial \mathrm{E}_z(\vec{r},t)}{\partial x} = \mu(\vec{r},t)\frac{\partial \mathrm{H}_y(\vec{r},t)}{\partial t} + \frac{\partial \mu(\vec{r},t)}{\partial t}\mathrm{H}_y(\vec{r},t) \tag{A8}$$

$$\frac{\partial \mathrm{H}_y(\vec{r},t)}{\partial x} - \frac{\partial \mathrm{H}_x(\vec{r},t)}{\partial y} = \varepsilon(\vec{r},t)\frac{\partial \mathrm{E}_z(\vec{r},t)}{\partial t} + \frac{\partial \varepsilon(\vec{r},t)}{\partial t}\mathrm{E}_z(\vec{r},t) \tag{A9}$$

According to Fig. A2, the discrete electric field and magnetic field can be written as

$$\mathrm{H}_x^{n+\frac{1}{2}}(i,j+\tfrac{1}{2})=\frac{\mu^{n-\frac{1}{2}}(i,j+\frac{1}{2})}{\mu^{n+\frac{1}{2}}(i,j+\frac{1}{2})}\mathrm{H}_x^{n-\frac{1}{2}}(i,j+\tfrac{1}{2})-\frac{\Delta t}{\mu^{n+\frac{1}{2}}(i,j+\frac{1}{2})}\frac{\mathrm{E}_z^{n}(i,j+1)-\mathrm{E}_z^{n}(i,j)}{\Delta y}\quad\text{(A10)}$$

$$\mathrm{H}_y^{n+\frac{1}{2}}(i+\tfrac{1}{2},j)=\frac{\mu^{n-\frac{1}{2}}(i+\frac{1}{2},j)}{\mu^{n+\frac{1}{2}}(i+\frac{1}{2},j)}\mathrm{H}_y^{n-\frac{1}{2}}(i+\tfrac{1}{2},j)+\frac{\Delta t}{\mu^{n+\frac{1}{2}}(i+\frac{1}{2},j)}\frac{\mathrm{E}_z^{n}(i+1,j)-\mathrm{E}_z^{n}(i,j)}{\Delta x}\quad\text{(A11)}$$

$$\mathrm{E}_z^{n+1}(i,j)=\frac{\varepsilon^{n}(i,j)}{\varepsilon^{n+1}(i,j)}\mathrm{E}_z^{n}(i,j)$$

$$+\frac{\Delta t}{\varepsilon^{n+1}(i,j)}\left(\frac{\mathrm{H}_y^{n+\frac{1}{2}}(i+\frac{1}{2},j)-\mathrm{H}_y^{n+\frac{1}{2}}(i-\frac{1}{2},j)}{\Delta x}-\frac{\mathrm{H}_x^{n+\frac{1}{2}}(i,j+\frac{1}{2})-\mathrm{H}_x^{n+\frac{1}{2}}(i,j-\frac{1}{2})}{\Delta y}\right)\quad\text{(A12)}$$

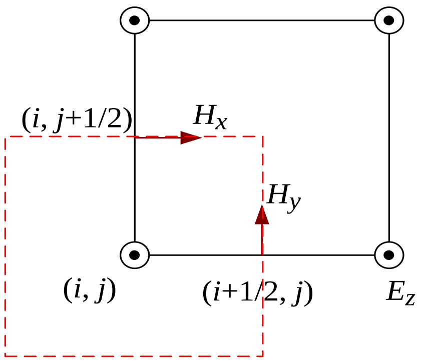

Fig. A2. TM wave Yee cell.

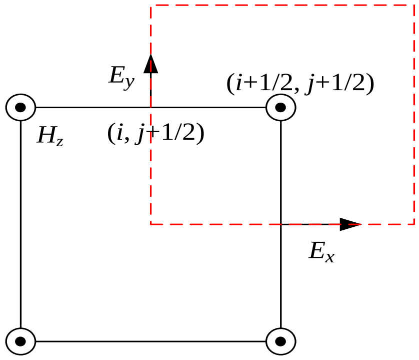

Fig. A3. TE wave Yee cell.

For two-dimensional photonic time crystal (TE wave), $\partial/\partial z=0$, Eq. (A1) and Eq. (A2) can be written as

$$\frac{\partial \mathrm{H}_z(\vec{r},t)}{\partial y}=\varepsilon(\vec{r},t)\frac{\partial \mathrm{E}_x(\vec{r},t)}{\partial t}+\frac{\partial\varepsilon(\vec{r},t)}{\partial t}\mathrm{E}_x(\vec{r},t)\qquad\text{(A13)}$$

$$-\frac{\partial \mathrm{H}_z(\vec{r},t)}{\partial x}=\varepsilon(\vec{r},t)\frac{\partial \mathrm{E}_y(\vec{r},t)}{\partial t}+\frac{\partial \varepsilon(\vec{r},t)}{\partial t}\mathrm{E}_y(\vec{r},t) \quad \text{(A14)}$$

$$\frac{\partial \mathrm{E}_y(\vec{r},t)}{\partial x}-\frac{\partial \mathrm{E}_x(\vec{r},t)}{\partial y}=-\mu(\vec{r},t)\frac{\partial \mathrm{H}_z(\vec{r},t)}{\partial t}-\frac{\partial \mu(\vec{r},t)}{\partial t}\mathrm{H}_z(\vec{r},t) \quad \text{(A15)}$$

According to Fig. A3, the discrete electric field and magnetic field can be written as

$$\mathrm{E}_{\mathrm{x}}^{\mathrm{n+1}}(i+\tfrac{1}{2},j)=\frac{\varepsilon^{n}(i+\frac{1}{2},j)}{\varepsilon^{n+1}(i+\frac{1}{2},j)}\mathrm{E}_{\mathrm{x}}^{\mathrm{n}}(i+\tfrac{1}{2},j)$$

$$+\frac{\Delta t}{\varepsilon^{n+1}(i+\frac{1}{2},j)}\frac{\mathrm{H}_{\mathrm{z}}^{\mathrm{n}+\frac{1}{2}}(i+\frac{1}{2},j+\frac{1}{2})-\mathrm{H}_{\mathrm{z}}^{\mathrm{n}+\frac{1}{2}}(i+\frac{1}{2},j-\frac{1}{2})}{\Delta y} \quad \text{(A16)}$$

$$\mathrm{E}_{\mathrm{y}}^{\mathrm{n+1}}(i,j+\tfrac{1}{2})=\frac{\varepsilon^{n}(i,j+\frac{1}{2})}{\varepsilon^{n+1}(i,j+\frac{1}{2})}\mathrm{E}_{\mathrm{y}}^{\mathrm{n}}(i,j+\tfrac{1}{2})$$

$$+\frac{\Delta t}{\varepsilon^{n+1}(i,j+\frac{1}{2})}\frac{\mathrm{H}_{\mathrm{z}}^{\mathrm{n}+\frac{1}{2}}(i+\frac{1}{2},j+\frac{1}{2})-\mathrm{H}_{\mathrm{z}}^{\mathrm{n}+\frac{1}{2}}(i-\frac{1}{2},j+\frac{1}{2})}{\Delta x} \quad \text{(A17)}$$

$$\mathrm{H}_{\mathrm{z}}^{\mathrm{n}+\frac{1}{2}}(i+\tfrac{1}{2},j+\tfrac{1}{2})=\frac{\mu^{n-\frac{1}{2}}(i+\frac{1}{2},j+\frac{1}{2})}{\mu^{\mathrm{n}+\frac{1}{2}}(i+\frac{1}{2},j+\frac{1}{2})}\mathrm{H}_{\mathrm{z}}^{\mathrm{n}-\frac{1}{2}}(i+\tfrac{1}{2},j+\tfrac{1}{2})$$

$$-\frac{\Delta t}{\mu^{n+\frac{1}{2}}(i+\frac{1}{2},j+\frac{1}{2})}\left(\frac{\mathrm{E}_{\mathrm{y}}^{\mathrm{n}}(i+1,j+\frac{1}{2})-\mathrm{E}_{\mathrm{y}}^{\mathrm{n}}(i,j+\frac{1}{2})}{\Delta x}-\frac{\mathrm{E}_{\mathrm{x}}^{\mathrm{n}}(i+\frac{1}{2},j+1)-\mathrm{E}_{\mathrm{x}}^{\mathrm{n}}(i+\frac{1}{2},j)}{\Delta y}\right) \quad \text{(A18)}$$

A3. Three-dimensional photonic time crystal

For three-dimensional photonic time crystal, Eq. (A1) and Eq. (A2) can be written as

$$\frac{\partial \mathrm{H}_z(\vec{r},t)}{\partial y}-\frac{\partial \mathrm{H}_y(\vec{r},t)}{\partial z}=\varepsilon(\vec{r},t)\frac{\partial \mathrm{E}_x(\vec{r},t)}{\partial t}+\frac{\partial \varepsilon(\vec{r},t)}{\partial t}\mathrm{E}_x(\vec{r},t) \quad \text{(A19)}$$

$$\frac{\partial \mathrm{H}_x(\vec{r},t)}{\partial z}-\frac{\partial \mathrm{H}_z(\vec{r},t)}{\partial x}=\varepsilon(\vec{r},t)\frac{\partial \mathrm{E}_y(\vec{r},t)}{\partial t}+\frac{\partial \varepsilon(\vec{r},t)}{\partial t}\mathrm{E}_y(\vec{r},t) \quad \text{(A20)}$$

$$\frac{\partial \mathrm{H}_y(\vec{r},t)}{\partial x}-\frac{\partial \mathrm{H}_x(\vec{r},t)}{\partial y}=\varepsilon(\vec{r},t)\frac{\partial \mathrm{E}_z(\vec{r},t)}{\partial t}+\frac{\partial \varepsilon(\vec{r},t)}{\partial t}\mathrm{E}_z(\vec{r},t) \quad \text{(A21)}$$

$$\frac{\partial \mathrm{E}_z(\vec{r},t)}{\partial y}-\frac{\partial \mathrm{E}_y(\vec{r},t)}{\partial z}=-\mu(\vec{r},t)\frac{\partial H_x(\vec{r},t)}{\partial t}-\frac{\partial \mu(\vec{r},t)}{\partial t}\mathrm{H}_x(\vec{r},t) \quad \text{(A22)}$$

$$\frac{\partial \mathrm{E}_x(\vec{r},t)}{\partial z}-\frac{\partial \mathrm{E}_z(\vec{r},t)}{\partial x}=-\mu(\vec{r},t)\frac{\partial H_y(\vec{r},t)}{\partial t}-\frac{\partial \mu(\vec{r},t)}{\partial t}\mathrm{H}_y(\vec{r},t) \quad \text{(A23)}$$

$$\frac{\partial \mathrm{E}_y(\vec{r},t)}{\partial x} - \frac{\partial \mathrm{E}_x(\vec{r},t)}{\partial y} = -\mu(\vec{r},t)\frac{\partial H_z(\vec{r},t)}{\partial t} - \frac{\partial \mu(\vec{r},t)}{\partial t}\mathrm{H}_z(\vec{r},t) \qquad \text{(A24)}$$

According to Fig. A4, the discrete electric field and magnetic field can be written as

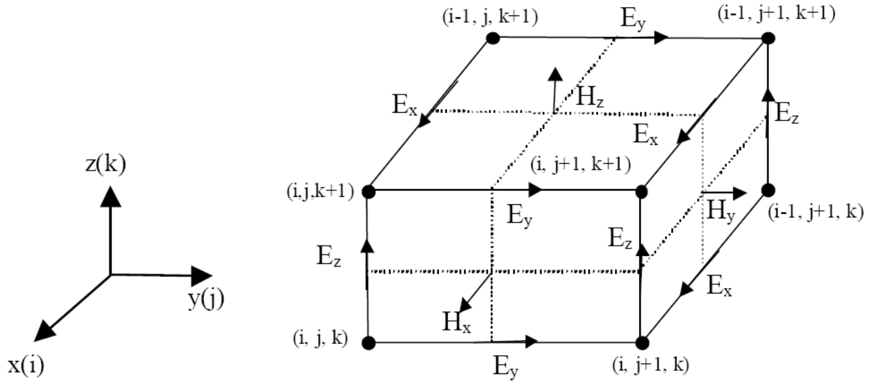

Fig. A4. Three-dimensional Yee cell.

$$\mathrm{E}_x^{n+1}(i+\tfrac{1}{2},j,k) = \frac{\varepsilon^n(i+\tfrac{1}{2},j,k)}{\varepsilon^{n+1}(i+\tfrac{1}{2},j,k)}\mathrm{E}_x^{n}(i+\tfrac{1}{2},j,k)$$

$$+\frac{\Delta t}{\varepsilon^{n+1}(i+\tfrac{1}{2},j,k)}\frac{\mathrm{H}_z^{n+\frac{1}{2}}(i+\tfrac{1}{2},j+\tfrac{1}{2},k)-\mathrm{H}_z^{n+\frac{1}{2}}(i+\tfrac{1}{2},j-\tfrac{1}{2},k)}{\Delta y}$$

$$-\frac{\Delta t}{\varepsilon^{n+1}(i+\tfrac{1}{2},j,k)}\frac{\mathrm{H}_y^{n+\frac{1}{2}}(i+\tfrac{1}{2},j,k+\tfrac{1}{2})-\mathrm{H}_y^{n+\frac{1}{2}}(i+\tfrac{1}{2},j,k-\tfrac{1}{2})}{\Delta z} \qquad \text{(A25)}$$

$$\mathrm{E}_y^{n+1}(i,j+\tfrac{1}{2},k) = \frac{\varepsilon^n(i,j+\tfrac{1}{2},k)}{\varepsilon^{n+1}(i,j+\tfrac{1}{2},k)}\mathrm{E}_x^{n}(i,j+\tfrac{1}{2},k)$$

$$+\frac{\Delta t}{\varepsilon^{n+1}(i,j+\tfrac{1}{2},k)}\frac{\mathrm{H}_x^{n+\frac{1}{2}}(i,j+\tfrac{1}{2},k+\tfrac{1}{2})-\mathrm{H}_x^{n+\frac{1}{2}}(i,j+\tfrac{1}{2},k-\tfrac{1}{2})}{\Delta z}$$

$$-\frac{\Delta t}{\varepsilon^{n+1}(i,j+\tfrac{1}{2},k)}\frac{\mathrm{H}_z^{n+\frac{1}{2}}(i+\tfrac{1}{2},j+\tfrac{1}{2},k)-\mathrm{H}_z^{n+\frac{1}{2}}(i-\tfrac{1}{2},j+\tfrac{1}{2},k)}{\Delta x} \qquad \text{(A26)}$$

$$\mathrm{E}_z^{n+1}(i,j,k+\tfrac{1}{2}) = \frac{\varepsilon^n(i,j,k+\tfrac{1}{2})}{\varepsilon^{n+1}(i,j,k+\tfrac{1}{2})}\mathrm{E}_z^{n}(i,j,k+\tfrac{1}{2})$$

$$+\frac{\Delta t}{\varepsilon^{n+1}(i,j,k+\tfrac{1}{2})}\frac{\mathrm{H}_y^{n+\frac{1}{2}}(i+\tfrac{1}{2},j,k+\tfrac{1}{2})-\mathrm{H}_y^{n+\frac{1}{2}}(i-\tfrac{1}{2},j,k+\tfrac{1}{2})}{\Delta x}$$

$$-\frac{\Delta t}{\varepsilon^{n+1}(i,j,k+\tfrac{1}{2})}\frac{\mathrm{H}_x^{n+\frac{1}{2}}(i,j+\tfrac{1}{2},k+\tfrac{1}{2})-\mathrm{H}_x^{n+\frac{1}{2}}(i,j-\tfrac{1}{2},k+\tfrac{1}{2})}{\Delta y} \qquad \text{(A27)}$$

$$\mathrm{H}_{\mathrm{x}}^{\mathrm{n}+\frac{1}{2}}(i,j+\tfrac{1}{2},k+\tfrac{1}{2})=\frac{\mu^{n-\frac{1}{2}}(i,j+\frac{1}{2},k+\frac{1}{2})}{\mu^{n+\frac{1}{2}}(i,j+\frac{1}{2},k+\frac{1}{2})}\mathrm{H}_{\mathrm{x}}^{\mathrm{n}-\frac{1}{2}}(i,j+\tfrac{1}{2},k+\tfrac{1}{2})$$

$$-\frac{\Delta t}{\mu^{n+\frac{1}{2}}(i,j+\frac{1}{2},k+\frac{1}{2})}\frac{\mathrm{E}_{\mathrm{z}}^{\mathrm{n}}(i,j+1,k+\frac{1}{2})-E_{\mathrm{z}}^{\mathrm{n}}(i,j,k+\frac{1}{2})}{\Delta y}$$

$$+\frac{\Delta t}{\mu^{n+\frac{1}{2}}(i,j+\frac{1}{2},k+\frac{1}{2})}\frac{\mathrm{E}_{\mathrm{y}}^{\mathrm{n}}(i,j+\frac{1}{2},k+1)-E_{\mathrm{y}}^{\mathrm{n}}(i,j+\frac{1}{2},k)}{\Delta z} \qquad \text{(A28)}$$

$$\mathrm{H}_{\mathrm{y}}^{\mathrm{n}+\frac{1}{2}}(i+\tfrac{1}{2},j,k+\tfrac{1}{2})=\frac{\mu^{n-\frac{1}{2}}(i+\frac{1}{2},j,k+\frac{1}{2})}{\mu^{n+\frac{1}{2}}(i+\frac{1}{2},j,k+\frac{1}{2})}\mathrm{H}_{\mathrm{y}}^{\mathrm{n}-\frac{1}{2}}(i+\tfrac{1}{2},j,k+\tfrac{1}{2})$$

$$-\frac{\Delta t}{\mu^{n+\frac{1}{2}}(i+\frac{1}{2},j,k+\frac{1}{2})}\frac{\mathrm{E}_{\mathrm{x}}^{\mathrm{n}}(i+\frac{1}{2},j,k+1)-E_{\mathrm{x}}^{\mathrm{n}}(i+\frac{1}{2},j,k)}{\Delta z}$$

$$+\frac{\Delta t}{\mu^{n+\frac{1}{2}}(i+\frac{1}{2},j,k+\frac{1}{2})}\frac{\mathrm{E}_{\mathrm{z}}^{\mathrm{n}}(i+1,j,k+\frac{1}{2})-E_{\mathrm{z}}^{\mathrm{n}}(i,j,k+\frac{1}{2})}{\Delta x} \qquad \text{(A29)}$$

$$\mathrm{H}_{\mathrm{z}}^{\mathrm{n}+\frac{1}{2}}(i+\tfrac{1}{2},j+\tfrac{1}{2},k)=\frac{\mu^{n-\frac{1}{2}}(i+\frac{1}{2},j+\frac{1}{2},k)}{\mu^{n+\frac{1}{2}}(i+\frac{1}{2},j+\frac{1}{2},k)}\mathrm{H}_{\mathrm{z}}^{\mathrm{n}-\frac{1}{2}}(i+\tfrac{1}{2},j+\tfrac{1}{2},k)$$

$$-\frac{\Delta t}{\mu^{n+\frac{1}{2}}(i+\frac{1}{2},j+\frac{1}{2},k)}\frac{\mathrm{E}_{\mathrm{y}}^{\mathrm{n}}(i+1,j+\frac{1}{2},k)-E_{\mathrm{y}}^{\mathrm{n}}(i,j+\frac{1}{2},k)}{\Delta x} \qquad \text{(A30)}$$

References

1. Yee, K. Numerical solution of initial boundary value problems involving Maxwell's equations in isotropic media. *IEEE Transactions on antennas and propagation*. ***14,*** 302-307 (1966).
